# Individualized follow up programme and early discharge in term neonates

**DOI:** 10.1186/1824-7288-40-70

**Published:** 2014-07-15

**Authors:** Maria Pia De Carolis, Carmen Cocca, Elisabetta Valente, Serafina Lacerenza, Serena Antonia Rubortone, Antonio Alberto Zuppa, Costantino Romagnoli

**Affiliations:** 1 Department of Paediatrics, Division of Neonatology, Catholic University of Sacred Heart, Largo A. Gemelli, 8, Rome 00168, Italy

**Keywords:** Early discharge, Follow-up, Neonate, Hyperbilirubinemia, Weight loss, Rehospitalization

## Abstract

**Background:**

Early discharge of mother/neonate dyad has become a common practice, and its effects are measured by readmission rates. We evaluated the safety of early discharge followed by an individualized Follow-up programme and the efficacy in promoting breastfeeding initiation and duration.

**Methods:**

During a nine-month period early discharge followed by an early targeted Follow-up was carried out in term neonates in the absence of weight loss <10% or hyperbilirubinaemia at risk of treatment. Follow-up visits were performed at different timepoints with a specific flow-chart according to both bilirubin levels and weight loss at discharge.

**Results:**

During the study period early discharge was performed in 419 neonates and Follow-up was carried out in 408 neonates (97.4%). No neonates required readmission for hyperbilirubinaemia and dehydration during the first 28 days of life. Breastfeeding rate was 90.6%, 75.2%, 41.5% at 30, 90 and 180 days of life, respectively. A six-month phone interview was performed for 383 neonates (93.8%) and satisfaction of parents about early discharge was high in 345 cases (90.1%).

**Conclusions:**

Early discharge in association with an individualized Follow-up programme resulted safe for the neonate and effective for breastfeeding initation and duration.

## Background

Early discharge (ED) of healthy late preterm and full term newborn infants has become a common practice because of current social and economic necessities [1,2]. The average length of stay of the mother-infant dyad after delivery declined steadily from 1970 until the mid-1990s [3,4] and in 1995, the American Academy of Pediatrics (AAP) defined early and very early discharge as stays of 48 and 24 hours, respectively, after uncomplicated vaginal delivery [5].

However, even if this practice has become more and more widespread, there was an increased rate of early readmission post discharge for jaundice, feeding problems, excessive weight loss, dehydration and hypernatremia [6-9]. For this reason, it is recommended that early discharged neonates should be evaluated at home or preferably in hospital within 48 hours after discharge [10-12].

The aim of the present study was to evaluate the safety of ED with an individualized Follow-up (FU) programme, according to both bilirubin levels and degree of weight loss (WL) at the time of discharge. Moreover, the efficacy in promoting breastfeeding and its duration was evaluated.

## Materials and methods

This observational study was conducted in the newborn nursery of the Catholic University of Sacred Heart of Rome during a nine-month period. During this period, an ED programme followed by an early targeted FU was carried out. Discharge to home was defined “early” when after 2-night stay, the infant was aged 48-63 hours.

Infants were eligible for enrolment if they were managed exclusively in the newborn nursery, if they were ≥37 weeks’ gestational age (GA) and if their birth weight (BW) was ≥2500 g. GA was based on postmenstrual date and early gestation prenatal sonographic findings.

The ED was not performed if at the scheduled time laboratory and/or other diagnostic tests were ongoing, in the presence of WL <10% or hyperbilirubinaemia at risk of treatment [13-15] and when the mother was not dischargeable at the same time.

None of the babies received drug therapy except for 1 mg vitamin K (Konakion, Roche Laboratories, Nutley, NJ, USA) intramuscularly soon after birth. Feeding was started at one hour of life, followed by breast or bottle feeding every 3 hours.

**Figure 1:**
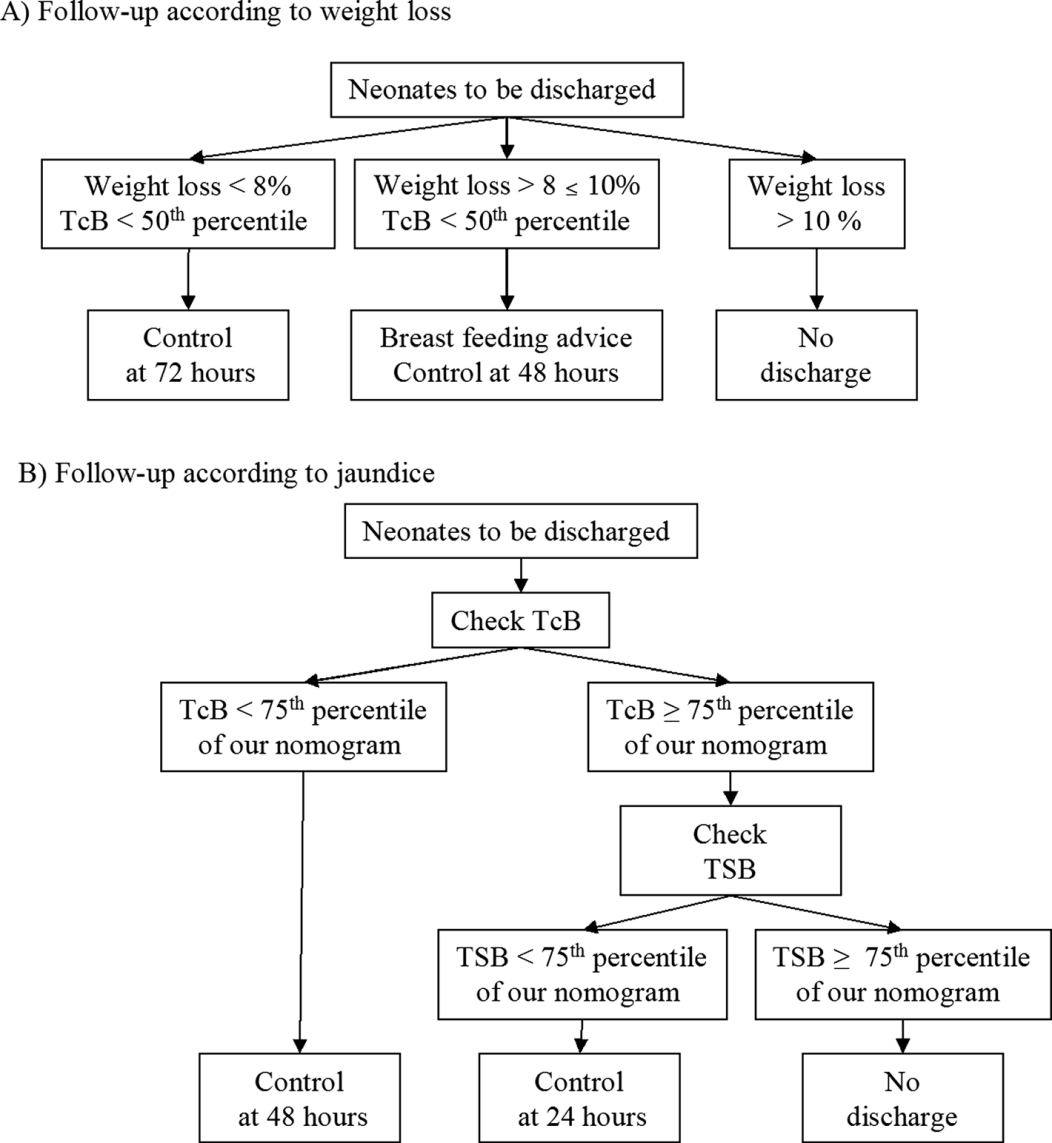
Follow-up according to weigth loss (A) and bilirubin level (B).

Breastfeeding practice was supported and promoted by a trained staff during the hospitalization and at FU visits, as well as the ability and confidence of the mother to provide adequate care of her infant was verified [16].All early discharged neonates were evaluated according to the FU programme planned at different times according to a flow-chart considering WL and bilirubin levels at discharge. (Figure 1).Infants were weighted daily during birth hospitalisation and weight change was defined as the difference between birth weight and each weight recorded subsequently, calculated as a percentage of birth weight. Neonates with WL <8% were evaluated 72 hours after discharge. In the presence of WL between 8 and 10%, neonates were evaluated 48 hours after discharge and their mothers were reinforced in infant care, particularly regarding infant feeding. Neonates with excess WL <10% were not discharged to home (Figure 1A).

Measurement of transcutaneous bilirubin (TcB) was performed in all neonates before discharge from the hospital. All determinations were made using BiliCheckTM ([BC] Respironics, Marietta, GA – USA).

In infants with pre-discharge TcB level ≥ 75^th^ percentile of nomogram in use in our Institution total serum bilirubin (TSB) level was measured [17,18]. The FU programme was performed according to TcB/TSB levels at discharge. Infants with pre-discharge TcB level <75^th^ percentile of the nomogram (Figure 1B) were discharged and examined 48 hours later; while those with TcB levels ≥75^th^ percentile but TSB levels <75^th^ percentile were examined 24 hours after discharge. Neonates with both TcB and TSB levels ≥75^th^ percentile were not early discharged (Figure 1B).

Infants with WL < 8%, without jaundice (TcB <50^th^ percentile) were checked 72 hours after discharge.

At the first FU visit the following features were evaluated: weight and weight variation compared to discharge and birth, hydration, TcB/TSB levels, general health, feeding pattern, and any new problem occurred during the home stay.

After the first FU control, neonates without hyperbilirubinaemia with physiological growth (or total WL <10%) were addressed to medical home service. On the opposite, a second FU visit was planned in the presence of a further WL and/or an increase of bilirubin level. Particularly for neonates with a further WL (<8%), a new control after 48 hours was planned. Neonates with hyperbilirubinemia were examined at different timepoints according to bilirubin level and need for treatment [19].

In order to evaluate the safety of ED and FU programme and to know the parents’ feedback, a phone interview at six months of age was performed. The following informations were collected: breastfeeding duration and reasons for its interruption; presence of diseases not diagnosed during the FU time; rate of emergency department visits or hospital readmission during the first month of life; family satisfaction about the ED.

### Statistical analysis

Statistical analysis was performed using “Stata Statistical Software: Release 12” (StataCorp LP, College Station, Tx). Continuous variables were presented as mean ± standard deviation or median (interquartile range) and categorical variables as number (percentage).

Univariate statistical analysis was performed using using Student’s “*t*” test for continuous variables, and Fisher’s exact test for categorical variables P < 0.05 was considered significant.

## Results

**Figure 2:**
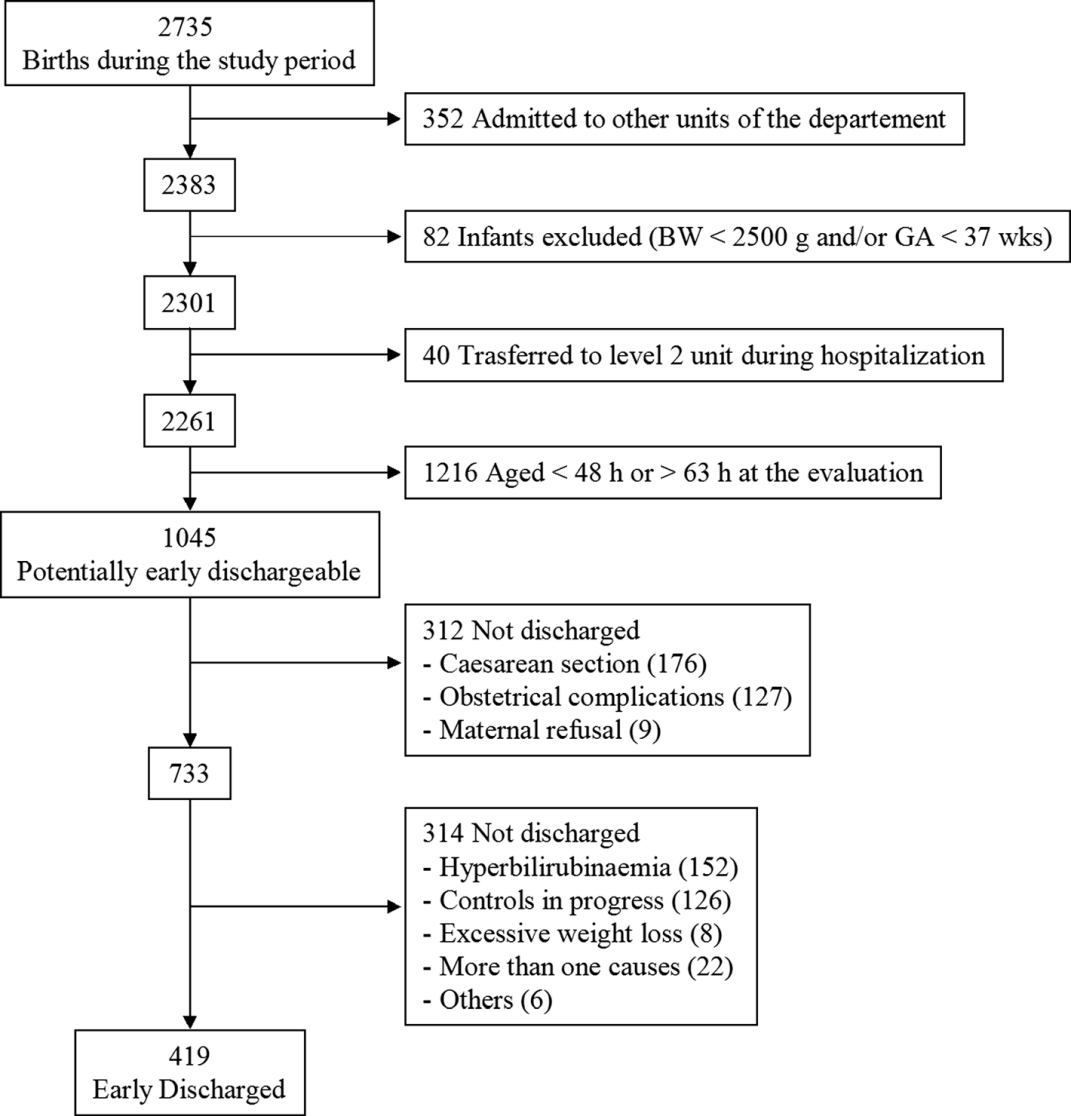
Flow diagram for inclusion.

During the study period, a total of 2735 neonates were born. Of them, 1690 were excluded because of admission to other units of the Pediatric Department immediately after birth (N = 352), GA <37 weeks and/or BW <2500 g (N = 82), transfer to other units during hospitalization (N = 40), age <48 or <63 hours after 2-night stay (N = 1216). Among the remaining 1045 infants potentially early dischargeable, 312 were not early discharged because of maternal causes (c-section, N = 176; obstetrical complications, N = 127; maternal refusal, N = 9). Early discharge was not performed in 314 cases for neonatal causes: 152 (48.4%) neonates were not discharged for hyperbilirubinaemia and 8 (2.5%) for WL <10%. In addition, also 126 (40.1%) neonates were not early discharged due to clinical and/or instrumental controls in progress, such as screening test for maternal bacterial or viral infection (N = 13) and screening or clinical observation for the prevention of group B streptococcal disease (N = 26), instrumental examination (N = 30), laboratory tests or specialistic evaluations (N = 32) or presence of abnormalities at clinical examination requiring continued hospitalization (N = 25). In 28 cases ED was not performed for the presence of more than one cause or for other causes. Consequently, 419 of 1045 infants potentially early dischargeable (40.1%) were early discharged (Figure 2). FU was performed in 408 of 419 neonates (97.4%); 11 infants were lost to FU because the parents refused to come back for an excessive home distance from hospital (N = 7) or for postpartum medical issues (N = 4), however they were evaluated by the medical home service.

Table 1 shows separately neonatal characteristics and data collected during the first FU visit performed at 24, 48 and 72 hours after ED. The first FU visit was performed 24 hours after discharge in 65 neonates (15.9%) for TcB ≥75^th^ percentile and TSB <75^th^ percentile. At 48 hours after discharge 195 neonates (47.8%) were evaluated, 3 of 195 (1.5%) for WL between 8 e 10% and 192 (98.5%) for TcB <75^th^ percentile at discharge. The remaining 148 neonates (36.3%) were evaluated 72 hours after discharge.

**Table 1:** Clinical characteristics of neonates early discharged in relation to the first Follow-up (FU)

**Timing of FU**	**24 hours (N = 65)**	**48 hours (N = 195)**	**72 hours (N = 148)**
Gestational age (wks)*	39.3 ± 1.1 (37-41)	39.4 ± 1.2 (37-42)	39.6 ± 1 (37-42)
Birth Weight (g)*	3326 ± 390 (2770-4650)	3379 ± 354 (2640-4440)	3384 ± 350 (2610-4440)
Hours of life at FU*	81.7 ± 5.3	105.8 ± 5.9	131.1 ± 8.4
Weight loss at discharge (% of birth weigth)*	4.3 ± 1.9 (1-8.9)	4.2 ± 2 (0-8.8)	4.2 ± 1.9 (0-9)
Weight loss at FU (% of birth weigth)*	4.0 ± 2.4 (0-9.9)	3.3 ± 2.5 (0-11)	2 ± 2.4 (0-13)
Recovery of birth weight at FU, n (%)	4 (6.2)	36 (18.4)	56 (37.8)
TcB at discharge (mg/dl)*	11.3 ± 1.6 (6.5-14.2)	8.7 ± 1.8 (1.7-13.3)	8.2 ± 2.2 (2.6-11.2)
TcB at FU (mg/dl)*	10.4 ± 3 (4.6-14.4)	8.7 ± 2.9 (1.7-15.4)	8 ± 3.3 (1.1-16.4)
TSB evaluation at FU, n (%)	46 (70.8)	34 (17.4)	11 (7.4)
TSB level at FU (mg/dl)*	10.7 ± 2.2 (5.7-15.2)	10.8 ± 2.6 (6-17.4)	9.9 ± 2.4 (4.6-12)
Exclusive breast feeding, n (%)	45 (69.3)	106 (54.3)	96 (64.9)
Breast + formula feeding, n (%)	19 (29.2)	82 (42.1)	45 (30.4)
Formula feeding, n (%)	1 (1.5)	1 (3.6)	7 (4.7)

Values expressed as: *mean ± DS (range).

None of the neonates evaluated at 24 hours showed further WL, in fact the WL was 4±2.4% at FU and 4.3±1.9% at discharge; only four neonates regained their BW. TcB levels were measured in all neonates and in 46 cases (70.7%) also TSB evaluation was needed and TSB levels were 10.7±2.2 mg/dl. Exclusive breastfeeding was noted in 45 neonates (69.3%), both breast and bottle feeding in 19 (29.2%), while one baby (1.5%) was exclusively formula fed.

In all neonates evaluated at 48 hours the WL was significantly lower than the one showed at discharge (4.2±2 vs 3.3±2.5; p <0.0001); 36 neonates (18.4%) had regained their BW. TcB levels were measured in all neonates, showing similar levels compared to those at discharge. However in 34 cases (17.4%) TSB evaluation was needed, showing mean values of 10.8±2.6 mg/dl. Exclusive breastfeeding was noted in 109 neonates (55.9%), both breast and bottle feeding in 85 (43.6%), and one neonate was exclusively formula fed (0.5%).

In all neonates evaluated at 72 hours the WL was significantly lower than that documented at discharge (4.2±1.9 vs 2±2.4 p < 0.0001), in fact 56 (18.4%) neonates regained their BW. TcB levels were measured in all neonates and only 11 cases (7.4%) needed TSB evaluation. Exclusive breastfeeding was noted in 96 neonates (64.9%), both breast and bottle feeding in 45 (30.4%), while 7 neonates (4.7%) were exclusively formula fed.

**Figure 3:**
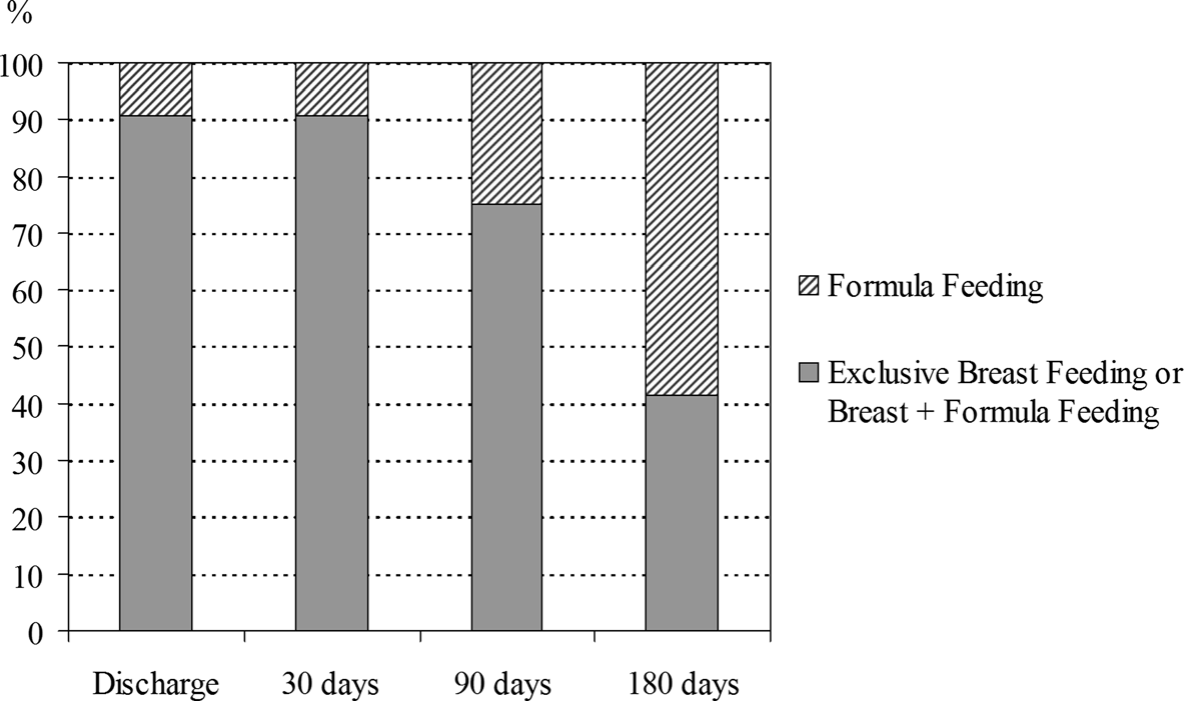
Feeding during the first 180 days of life.

A second FU visit was performed only in 42 of 408 (10.3%) neonates. In Table 2 the number and the causes of neonates requiring a second FU visit are reported. Seven neonates needed more than two evaluations because of hyperbilirubinaemia.A six-month phone interview was performed for 383 neonates (93.8%). A progressive reduction in breastfeeding rate was recorded (Figure 3). Breastfeeding rate was 90.6%, 75.2%, 41.5% at 30, 90 and 180 days of life respectively. No neonate required hospital readmission within the first month of life, only two neonates were conducted to the emergency department, one for infantile colic and one for erythema toxicum neonatorum. All infants were evaluated once or more times a month by a paediatrician.

**Table 2:** Number of neonates requiring check <1 at Follow-up (FU), separately respect to the first FU and causes

	**24 hours (N = 65)**	**48 hours (N = 195)**	**72 hours (N = 148)**
Second FU check	22 (33,8)	16 (8,2)	4 (2,7)
Hyperbilirubinaemia	13 (59,1)	8 (50)	-
Weight loss	9 (40,9)	8 (50)	4 (100)
FU check < 2	5 (7,7)	2 (1)	-

Parents’ satisfaction about ED was high in 345 cases (90.1%), medium in 30 cases (7.8%) and low in 8 cases (2.1%). A positive parental feedback was significantly influenced by maternal age (higher satisfaction in women aged <35 vs ≤ 35 years, p < 0.001) and parity (higher satisfaction in multiparous vs primiparous women, p < 0.05). Problems with transportation (N = 15, 39.4%), short time learning to care the infant (N = 16, 42.2%) and fatigue (N = 7, 18.4%) was the main causes for low or medium parental feedback.

## Discussion

The results of this study show that ED in association with individualized FU programme based on the evaluation of the risk factors, such as bilirubin level and WL, resulted safe for the term neonate. Despite the early period of birth hospitalization, FU programme determined a good parental satisfaction, giving confidence to provide adequate neonatal care.

However in our study the overall prevalence of ED was only 40.1%, lower than the one reported in other studies on term neonates (83.5%-93.4%) or in studies including both term and late preterm infants (64%) [20]. This low prevalence rate was related to both maternal and neonatal causes.

Among maternal causes the prominent role was the presence of c-sections and postpartum obstetric complications, due to the high number of high risk pregnancies afferent to our hospital. As suggested by the AAP early discharge was not performed after c-section but only after uncomplicated vaginal delivery [5]. The lack of mother discharge did not allow the simultaneous discharge of the mother-infant dyad. As recently reviewed by Evans et al. [21], many analyses of the impact of ED restricted their attention to uncomplicated vaginal or c-section deliveries and lacked detailed control variables, especially measures of pregnancy complications. It’s well known that a longer postpartum hospital stay is required for the mother with complications during pregnancy and delivery, so that the results of the logistic model show that an increased number of complications during pregnancy increases the hospital length stay [21].

Among neonatal problems, hyperbilirubinaemia was the main reason contraindicating ED. During birth hospitalization, evaluation of risk factors for hyperbilirubinaemia, TcB/TSB measurements and use of hour-specific nomograms, as suggested by Romagnoli C et al. [17,18] performed a safe discharge, identifying newborn at risk for significant hyperbilirubinaemia. The safety of ED according to these parameters is confirmed by data of the FU, since no rehospitalization for hyperbilirubinaemia was recorded. In our experience, only a small number of neonates was not discharged because of an extreme WL (<10%). We think that this result was reached thanks to the good mothers’ ability on breastfeeding learned during childbirth courses and overall to the appropriate support by a trained staff on promotion and continuation of breastfeeding after delivery [22]. However, ED was not performed in 126 cases because laboratory and/or other diagnostic tests were still ongoing at the time of discharge. A better scheduling of clinical actions and more efficient services could hereafter increase the number of the infants early discharged. In addition non-critical laboratory test results could be communicated to the family and eventually to the medical home service after discharge. Moreover, an early contact with medical home service could facilitate the performance of non-urgent tests after discharge.

It is interesting to note that in our experience the rate of neonates receiving FU is 97.4%, higher compared to those reported by Galbraith et al. (56.2%) [23] and to a recent surveillance system of PRAMS (Pregnancy Risk Assessment Monitoring System) showing a prevalence between 51.5% and 78.6% [20]. Obstetrical and newborn nursery care by a trained staff and the trust in the relationship with parents during birth hospitalization, the easy access to FU service, the availability of health workers may have probably contributed to this high return to FU.

The personalized FU programme based on bilirubin level at discharge allowed the monitoring of the bilirubin trend after discharge [17,18,24]. Although the higher bilirubin levels at discharge required more frequent FU visits, the neonate after FU was safely sent to medical home service.

Also our personalized FU programme based on WL at discharge resulted safe, since there were no infants rehospitalized because of an excessive WL and dehydration. The support offered by a trained staff during both hospitalization and FU visits could reinforce maternal and family education regarding infant feeding. In addition, the care regarding breastfeeding initiation and promotion might have also improved the breastfeeding duration in our experience. The rate of breastfeeding was in fact 90.6% at the first month of life and 75.2% after three months.

The FU programme was free so that it was well accepted by the family, and was performed with health care staff already known by the parents. The presence of the same professional health care staff immediately after birth and at FU visits guaranteed continuity giving safety to the family. The continuous family support was probably also the main factor of parents satisfaction and of lack of infant readmission for parents anxiety. It is reported that the parental anxiety in the absence of real illness is the cause of readmission in 2% of cases [4].

A limit of the present study is represented by the high number of neonates potentially dischargeable but not discharged because of ongoing diagnostic tests at discharge.

Our future aim would be to increase the rate of ED looking for a greater connection with the basic health services providing further information about ED and individualized FU to the mothers during childbirth courses, also involving the fathers [25].

In conclusion, we think that the definition “early discharge” could be replaced by “appropriate discharge”, since each mother-infant dyad should be evaluated individually to determine the optimal time of both discharge and FU timing.

## Conclusions

Our study shows that ED in association with an individualized FU programme resulted safe for the neonate since there were no infants rehospitalized because of an excessive WL and dehydration. The breastfeeding rate resulted high during the study period thanks to the adequate care regarding breastfeeding initiation and promotion.

## Consent

Written informed consent was obtained from the patient’s guardian/parent/next of kin for the publication of this report and any accompanying images.

## Abbreviations

BW: Birth weight; ED: Early discharge; FU: Follow-up; GA: Gestational age; TcB: Transcutaneous bilirubin; TSB: Total serum bilirubin; WL: Weight loss.

## Competing interests

The authors declare that they have no competing interests.

## Authors’ contributions

CR and ZAA conceived the design of the study. EV and SL participated in the design of the study, performed the data collection. MPD and CC participated in coordination of the study and drafted the manuscript and the statistical analysis. SAR helped to draft the manuscript. All authors read and approved the final manuscript.

## References

[B1] Maisels MJ Kring E Length of stay, jaundice, and hospital readmission Pediatrics 1998 101 995 998 10.1542/peds.101.6.995 9606225

[B2] Mercier CE Barry SE Paul K Delaney TV Horbar JD Wasserman RC Berry P Shaw JS Improving newborn preventive services at the birth hospitalization: a collaborative, hospital-based quality-improvement project Pediatrics 2007 120 481 488 10.1542/peds.2007-0233 17766519

[B3] Datar A Sood N Impact of postpartum hospital-stay legislation on newborn length of stay, readmission, and mortality in California Pediatrics 2006 118 63 72 10.1542/peds.2005-3044 16818550

[B4] Oddie SJ Hammal D Richmond S Parker L Early discharge and readmission to hospital in the first month of life in the Northern Region of the UK during 1998: a case cohort study Arch Dis Child 2005 90 119 124 10.1136/adc.2003.040766 15665161 PMC1720274

[B5] American Academy of Pediatrics and American College of Obstetrics and Gynecology Postpartum and follow-up care Guidelines for Perinatal Care 4 1992 3 Elk Grove Village, IL: American Academy of Pediatrics; Washington, DC: American College of Obstetricians and Gynecologists 91 116

[B6] Braveman P Egerter S Pearl M Marchi K Miller C Problems associated with early discharge of newborn infants. Early discharge of newborns and mothers: a critical review of the literature Pediatrics 1995 96 716 726 7567337

[B7] Oddie SJ Craven V Deakin K Westman J Scally A Severe neonatal hypernatraemia: a population based study Arch Dis Child Fatal Neonatal Ed 2013 98 F384 F387 10.1136/archdischild-2012-302908 23512226

[B8] Young PC Korgenski K Buchi KF Early readmission of newborns in a large health care system Pediatrics 2013 131 e1538 e1544 10.1542/peds.2012-2634 23569092

[B9] Farhat R Rajab M Length of postnatal hospital stay in healthy newborns and re-hospitalization following early discharge N Am J Med Sci 2011 3 146 151 22540081 10.4297/najms.2011.3146 PMC3336902

[B10] American Academy of Pediatrics. Committee on Fetus and Newborn Hospital stay for healthy term newborns Pediatrics 1995 96 788 790 7567351

[B11] Yonemoto N Dowswell T Nagai S Mori R Schedules for home visits in the early postpartum period Cochrane Database Syst Rev 2013 23 7 10.1002/14651858.CD009326.pub2 23881661

[B12] Meara E Kotagal UR Atherton HD Lieu TA Impact of early newborn discharge legislation and early follow-up visits on infant outcomes in a state Medicaid population Pediatrics 2004 113 1619 1627 10.1542/peds.113.6.1619 15173482

[B13] American Academy of Pediatrics. Subcommittee on Hyperbilirubinemia Management of hyperbilirubinemia in the newborn infant 35 or more weeks of gestation Pediatrics 2004 114 297 316 15231951 10.1542/peds.114.1.297

[B14] Bhutani VK Stark AR Lazzeroni LC Poland R Gourley GR Kazmierczak S Meloy L Burgos AE Hall JY Stevenson DK Initial Clinical Testing Evaluation and Risk Assessment for Universal Screening for Hyperbilirubinemia Study Group Predischarge screening for severe neonatal hyperbilirubinaemia identifies infants who need phototherapy J Pediatr 2013 162 477 482 10.1016/j.jpeds.2012.08.022 23043681

[B15] De Luca D Carnielli VP Paollilo P Neonatal hyperbilirubinemia and early discharge from maternity ward Eur J Pediatr 2009 68 1025 1030 19277705 10.1007/s00431-009-0969-1

[B16] American Academy of Pediatrics. Committee on Fetus and Newborn Policy statement-Hospital stay for healthy term newborns Pediatrics 2010 125 405 40920100744

[B17] De Luca D Romagnoli C Tiberi E Zuppa AA Zecca E Skin bilirubin nomogram for the first 96 h of life in a European normal healthy newborn population, obtained with multiwavelength transcutaneous bilirubinometry Acta Paediatr 2008 97 146 150 10.1111/j.1651-2227.2007.00622.x 18254903

[B18] Romagnoli C Tiberi E Barone G Curtis MD Regoli D Paolillo P Picone S Anania S Finocchi M Cardiello V Giordano L Paolucci V Zecca E Development and validation of serum bilirubin nomogram to predict the absence of risk for severe hyperbilirubinaemia before discharge: a prospective, multicenter study Ital J Pediatr 2012 1 38 46 10.1186/1824-7288-38-6 PMC3298708 22296875

[B19] Bhutani VK Maisels MJ Stark AR Buonocore G Expert Committee for Severe Neonatal Hyperbilirubinemia; European Society for Pediatric Research; American Academy of Pediatrics Management of jaundice and prevention of severe neonatal hyperbilirubinemia in infants ≥ 35 weeks of gestation Neoanatology 2008 94 63 67 10.1159/000113463 18204221

[B20] Lansky A Barfield WD Marchi KS Egerter SA Galbraith AA Braveman PA Early postnatal care among healthy newborns in 19 states: pregnancy risk assessment monitoring system, 2000 Matern Child Health J 2006 10 277 284 10.1007/s10995-005-0050-2 16382330

[B21] Evans WN Garthwaite C Wei H The impact of early discharge laws on the health of newborns J Health Econ 2008 27 843 870 10.1016/j.jhealeco.2007.12.003 18308409

[B22] Martín-Iglesias S Del-Cura-González I Sanz-Cuesta T Arana-Cañedo Argüelles C Rumayor-Zarzuelo M Alvarez-de la Riva M Lloret-Sáez Bravo AM Férnandez-Arroyo RM Aréjula-Torres JL Aguado-Arroyo Ó Góngora-Maldonado F García-Corraliza M Sandoval-Encinas N Tomico-delRío M Cornejo-Gutiérrez AM Effectiveness of an implementation strategy for a breastfeeding guideline in Primary Care: cluster randomised trial BMC Fam Pract 2011 12 144 10.1186/1471-2296-12-144 22208800 PMC3339325

[B23] Galbraith AA Egerter SA Marchi KS Chavez G Braveman PA Newborn early discharge revisited: are California newborns receiving recommended postnatal services? Pediatrics 2003 111 364 371 10.1542/peds.111.2.364 12563065

[B24] Newman TB Liljestrand P Escobar GJ Combining clinical risk factors with serum bilirubin levels to predict hyperbilirubinemia in newborns Arch Pediatr Adolesc Med 2005 159 113 119 15699303 10.1001/archpedi.159.2.113

[B25] Carlander AK Edman G Christensson K Andolf E Wiklund I Contact between mother, child and partner and attitudes towards breastfeeding in relation to mode of delivery Sex Reprod Healthc 2010 1 27 34 10.1016/j.srhc.2009.10.001 21122593

